# Supplementary figures and images for: The First Record of Taiga Shrew in Lithuania
Source: Animals (Basel). 2025 Oct 24;15(21):3088. doi: 10.3390/ani15213088 (PMC12610762; doi:10.3390/ani15213088)

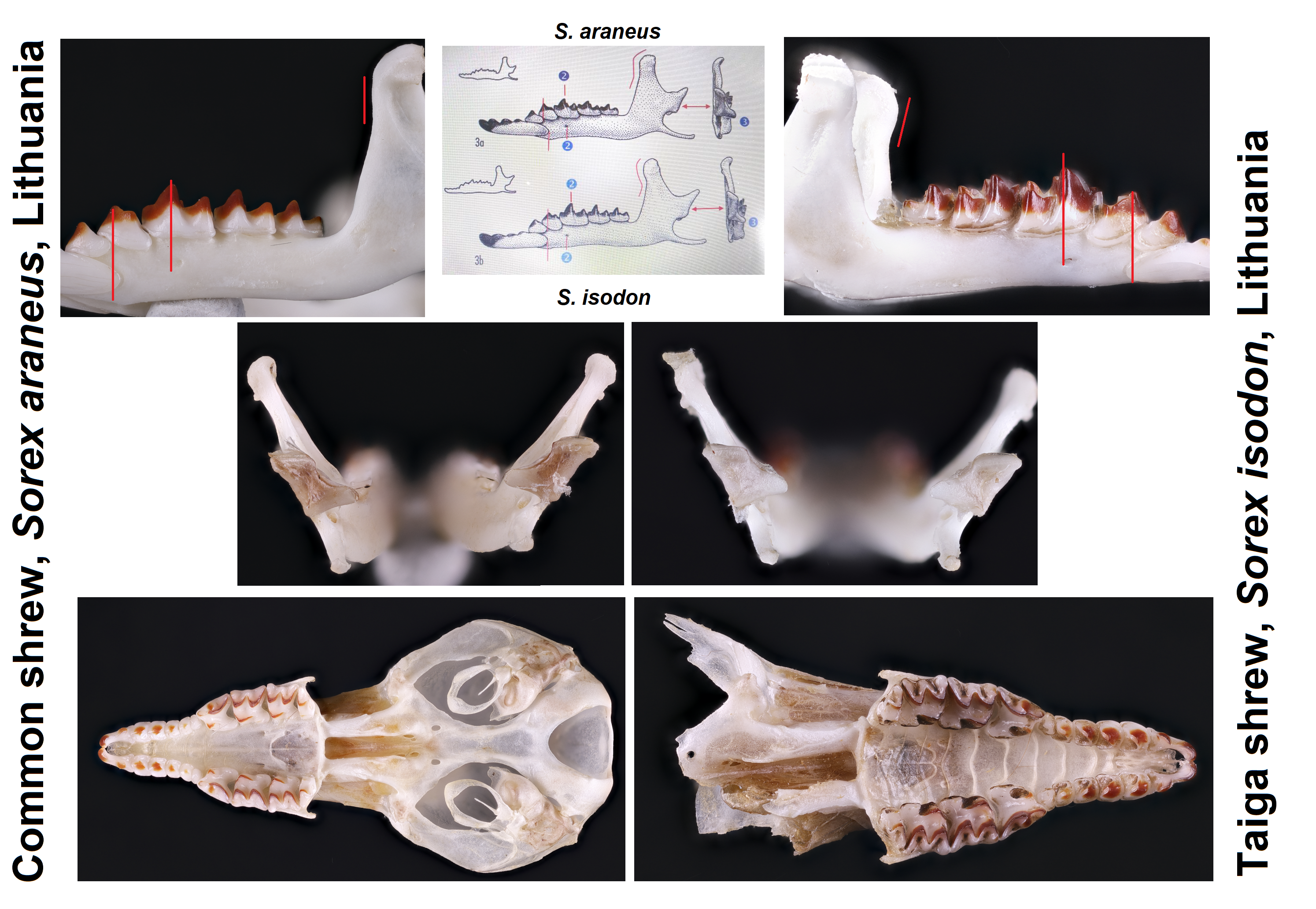

Supplement: Supplementary file 1 [file animals-15-03088-s001.zip › Figure S1.tif]
